# Prehospital thrombolytic treatment of high-risk acute pulmonary embolism

**DOI:** 10.1186/s13054-025-05465-w

**Published:** 2025-07-07

**Authors:** Hugo Lanz, Leonhard Binzenhöfer, Tom Verbelen, Andrea Stadlbauer, Daniele Camboni, Sebastian Zimmer, Georg Nickenig, Holger Thiele, Enzo Lüsebrink, the High-risk Investigator Group, Hugo Lanz, Leonhard Binzenhöfer, Tom Verbelen, Andrea Stadlbauer, Daniele Camboni, Sebastian Zimmer, Georg Nickenig, Holger Thiele, Enzo Lüsebrink, Federico Pappalardo, Frederic De Roeck, Christiaan Vrints, Aitor Uribarri, Jorge Moisés, Manel Sabate, Jeisson Osorio, Luis Chiscano-Camón, Jordi Riera, Carsten Skurk, Hannah Billig, Christoph Adler, Tobias Tichelbäcker, Peter M. Spieth, Lisa Crusius, Norman Mangner, Christian Jung, Dirk Westermann, Esther Tautz, Alexander Supady, Kirsten Krüger, Karl Toischer, Aschraf El-Essawi, Daniel Hoyer, Jörn Tongers, Jochen Dutzmann, Andreas Schäfer, Mostafa Salem, Derk Frank, Marvin Kriz, Benedikt Schrage, Benedikt Beer, Franz Haertel, Sven Möbius-Winkler, Nicolas Majunke, Tom Adriaenssens, Andreas Verstraete, Tomaz Goslar, Marko Noc, Tobias Graf, Ingo Eitel, Maike Knorr, Clemens Scherer, Laura Villegas Sierra, Nils Gade, Daniel Roden, Inas Saleh, Sabine Hoffmann, Michael Schomaker, Julia Höpler, Marie Kraft, Sebastian Kufner, Karsten Hug, Christian Hagl, Sven Peterss, Nikolaus Kneidinger, Carsten Hullermann, Jan Sackarnd, Alain Combes, Guido Tavazzi, Christof Schmid, Elizabete Terauda, Andrejs Erglis, Evija Camane, Santa Strazdina, Līga Vīduša, Oliver Borst, Helene Häberle, Walter S. Speidl, Robert Zilberszac, Robert H. G. Schwinger, Silvia Klinger, Antonia Wechsler

**Affiliations:** 1 Medizinische Klinik Und Poliklinik I, Klinikum Der Universität München, Munich, Germany and DZHK (German Center for Cardiovascular Research), Partner Site Munich Heart Alliance, Munich, Germany; 2 Department of Cardiac Surgery, University Hospitals Leuven, Louvain, Belgium; 3 Department of Cardiovascular Sciences, KU Louvain – University of Leuven, Louvain, Belgium; 4 Department of Cardiothoracic Surgery, University Medical Center Regensburg, Regensburg, Germany; 5 Medizinische Klinik Und Poliklinik II, Universitätsklinikum Bonn, Venusberg-Campus 1, 53127 Bonn, Germany; 6 Department of Internal Medicine/Cardiology and Leipzig Heart Science, Heart Center Leipzig at University of Leipzig, Leipzig, Germany

Acute pulmonary embolism (PE) is a prevalent cause of morbidity and mortality, with high-risk PE accounting for 5% of all cases and being associated with high mortality [1]. This subgroup is traditionally treated with guideline recommended systemic thrombolysis (SYS) [2]. Results from a recent international target trial emulation found surgical thrombectomy or percutaneous catheter-directed treatments (PCDT) may provide short-term survival benefit [3], and these treatments are reflected by guidelines as alternatives following failure of SYS [2]. While guidelines support SYS for suspected/confirmed PE-related cardiac arrest, evidence stems from small trials. Despite formidable prognosis, current guidelines lack management recommendations for this subgroup. Veno-arterial extracorporeal membrane oxygenation (VA-ECMO) may stabilize patients following circulatory collapse, and recent interest in PCDT has also grown. While no study has investigated optimal treatment upon admission following prehospital SYS, here we analyze different treatments in a preregistered subgroup originally excluded from a recently published high-risk PE study [3]. In this study, retrospective data from 991/1,060 high-risk PE patients treated between 2012 and 2022 were included in a target trial emulation designed to investigate in-hospital all-cause mortality with different treatment strategies (SYS, VA-ECMO, surgical thrombectomy, and PCDT), as described previously [3]. Here we investigated excluded patients treated with prehospital SYS having received: (1) no further treatment, (2) VA-ECMO (following prehospital SYS), (3) intrahospital SYS (additional SYS following admission), (4) surgical thrombectomy (following prehospital SYS), or (5) PCDT (following prehospital SYS).

Overall, 69 high-risk PE patients (49.3% male) with a median age of 54 years received prehospital SYS. 29/69 underwent prehospital SYS alone, 27/69 received VA-ECMO, 8/69 received additional intrahospital SYS, and 5/69 were treated with PCDT following hospital admission. 10/69 were escalated to a third treatment strategy (Fig. 1). Cardiac arrest had occurred in 88% by admission, with a median cardiopulmonary resuscitation duration of 55 min (VA-ECMO 45 min; intrahospital SYS 75 min). Intrahospital SYS patients exhibited most severe median admission Simplified Acute Physiology Score II (70.0), lowest pH levels (6.85), and highest lactate levels (20.0 mmol/L), while the PCDT group had more favorable admission values (admission pH 7.18, admission lactate 4.00 mmol/L) and suffered fewer cardiac arrests (2/5). Computed tomography angiography diagnosed PE in most patients (62.3%), with large between-group differences (PCDT 100% vs. intrahospital SYS 37.5%). Diagnosis on clinical suspicion alone was made in nearly 1/3 of all patients (62.5% in intrahospital SYS). Overall, in-hospital mortality was high (73.9%), with lower mortality rates following PCDT (40.0%) compared to VA-ECMO (77.8%) or intrahospital SYS (75.0%). Total one-year mortality climbed to 78.3%. Further, most major bleeding occurred with VA-ECMO (40.7%). Lastly, 12/18 survivors showed full neurologic recovery (Cerebral Performance Category 1) at discharge (PCDT 33.3%; intrahospital SYS 50%) (Tab. 1).

**Fig. 1:**
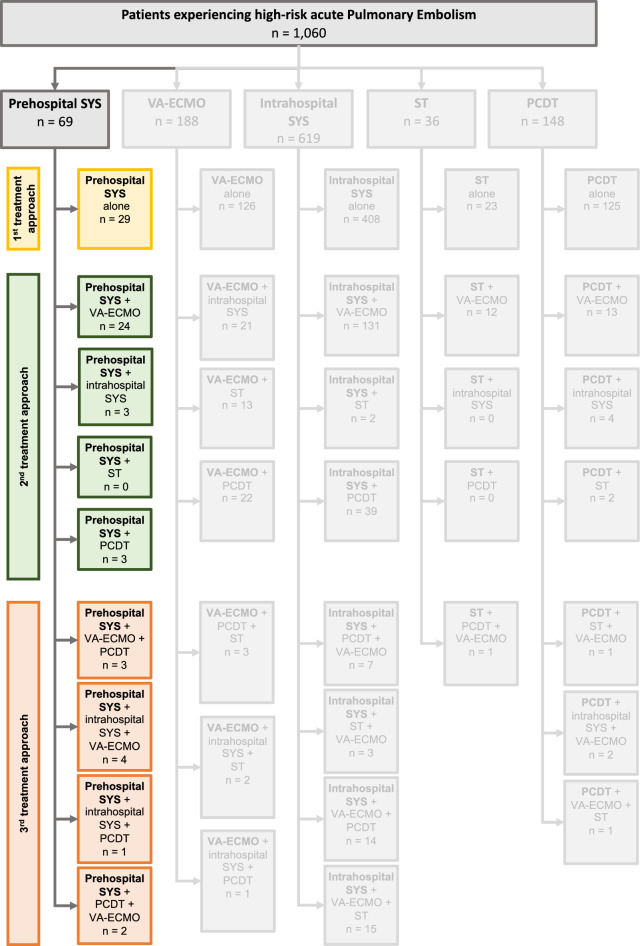
Overview of treatment approaches. PCDT, percutaneous catheter-directed treatment; ST, surgical thrombectomy; SYS, systemic thrombolysis; VA-ECMO, veno-arterial extracorporeal membrane oxygenation

**Table 1:** Characteristics and outcomes following prehospital systemic thrombolysis

Characteristics		Overall *n* = 69	Prehospital systemic thrombolysis *alone* (*n* = 29)	Prehospital systemic thrombolysis + VA-ECMO (*n* = 27)	Prehospital systemic thrombolysis + intrahospital systemic thrombolysis (*n* = 8)	Prehospital systemic thrombolysis + surgical thrombectomy (*n* = 0)	Prehospital systemic thrombolysis + catheter-directed treatment (*n* = 5)
Demographics							
Age at admission [years], median [IQR]		54.00 [42.60, 64.00]	58.00 [46.00, 68.00]	48.80 [35.65, 59.00]	48.50 [36.25, 56.50]	–	64.00 [58.00, 70.00]
Sex at birth [male], *n* (% [95% CI])		34 (49.3 [37.8, 60.8])	14 (48.3 [31.4, 65.6])	13 (48.1 [30.7, 66.0])	3 (37.5 [13.7, 69.4])	–	4 (80.0 [37.6, 96.4])
Morbidity at hospital admission							
Cardiac arrest, *n* (% [95% CI])		61 (88.4 [78.8, 94.0])	26 (89.7 [73.6, 96.4])	25 (92.6 [76.6, 97.9])	8 (100.0 [67.6, 100.0])	–	2 (40.0 [11.8, 76.9])
Cardiopulmonary resuscitation, *n* (% [95% CI])		61 (88.4 [78.8, 94.0])	27 (93.1 [78.0, 98.1])	24 (88.9 [71.9, 96.1])	8 (100.0 [67.6, 100.0])	–	2 (40.0 [11.8, 76.9])
CPR duration [min], median [IQR]		55.00 [30.00, 75.00]	55.00 [28.50, 60.00]	45.00 [30.00, 78.75]	75.00 [52.50, 92.50]	–	67.50 [58.75, 76.25]
Arterial lactate [mmol/L], median [IQR]*		14.50 [7.80, 17.65]	12.88 [6.10, 16.80]	15.00 [9.33, 17.98]	20.00 [15.05, 23.89]	–	4.00 [2.80, 9.70]
pH, median [IQR]*		6.91 [6.78, 7.15]	6.90 [6.78, 7.21]	6.89 [6.72, 7.11]	6.85 [6.79, 6.91]	–	7.18 [7.14, 7.30]
PaO_2_ [mmHg], median [IQR]*		94.30 [72.00, 155.75]	106.00 [76.25, 185.25]	81.00 [59.25, 110.50]	116.10 [78.55, 172.25]	–	113.50 [90.25, 162.75]
PaCO_2_ [mmHg], median [IQR]*		56.35 [39.70, 68.05]	56.35 [42.25, 69.75]	57.50 [40.45, 66.75]	57.90 [39.65, 71.05]	–	39.00 [37.25, 40.15]
SAPS II score at admission, median [IQR]		60.50 [44.00, 71.68]	61.00 [47.00, 85.00]	53.00 [36.75, 65.50]	70.00 [57.00, 71.90]	–	52.50 [36.00, 75.75]
SOFA score at admission, median [IQR]		12.00 [10.00, 14.00]	11.50 [8.75, 13.25]	13.00 [12.00, 14.00]	10.00 [10.00, 12.00]	–	11.00 [6.00, 12.00]
Diagnosis modality							
PE diagnosis made on CT scan, *n* (% [95% CI])		43 (62.3 [50.5, 72.8])	16 (55.2 [37.5, 71.6])	19 (70.4 [51.5, 84.1])	3 (37.5 [13.7, 69.4])	–	5 (100.0 [56.6, 100.0])
PE diagnosis made on pulmonary angiography, *n* (% [95% CI])		4 (5.8 [2.3, 14.0])	0 (0.0 [0.0, 11.7])	4 (14.8 [5.9, 32.5])	0 (0.0 [0.0, 32.4])	–	0 (0.0 [0.0, 43.4])
PE diagnosis based on high clinical probability, acute RV dysfunction, and the absence of other plausible causes, in accordance with the ESC guidelines, *n* (% [95% CI])		22 (31.9 [22.1, 43.6])	13 (44.8 [28.4, 62.5])	4 (14.8 [5.9, 32.5])	5 (62.5 [30.6, 86.3])	–	0 (0.0 [0.0, 43.4])
Mechanical circulatory support							
Venoarterial extracorporeal membrane oxygenation, *n* (% [95% CI])		33 (47.8 [36.5, 59.4])	0 (0.0 [0.0, 11.7])	27 (100.0 [87.5, 100.0])	4 (50.0 [21.5, 78.5])	–	2 (40.0 [11.8, 76.9])
Total duration of VA-ECMO treatment [h], median [IQR]		42.00 [9.75, 73.50]	-	52.50 [17.00, 87.00]	7.00 [4.00, 7.50]	–	27.00 [22.50, 31.50]
Outcome							
In-hospital all-cause mortality, *n* (% [95% CI])		51 (73.9 [62.5, 82.8])	22 (75.9 [57.9, 87.8])	21 (77.8 [59.2, 89.4])	6 (75.0 [40.9, 92.9])	–	2 (40.0 [11.8, 76.9])
1-month all-cause mortality, *n* (% [95% CI])		50 (72.5 [61.0, 81.6])	21 (72.4 [54.3, 85.3])	21 (77.8 [59.2, 89.4])	6 (75.0 [40.9, 92.9])	–	2 (40.0 [11.8, 76.9])
3-month all-cause mortality, *n* (% [95% CI])		52 (75.4 [64.0, 84.0])	22 (75.9 [57.9, 87.8])	21 (77.8 [59.2, 89.4])	6 (75.0 [40.9, 92.9])	–	3 (60.0 [23.1, 88.2])
1-year all-cause mortality, *n* (% [95% CI])		54 (78.3 [67.2, 86.4])	22 (75.9 [57.9, 87.8])	22 (81.5 [63.3, 91.8])	6 (75.0 [40.9, 92.9])	–	4 (80.0 [37.6, 96.4])
Total length of ICU stay [d], median [IQR]		3.00 [1.00, 10.00]	3.00 [1.00, 11.00]	3.00 [1.56, 10.00]	1.00 [1.00, 5.50]	–	4.00 [1.00, 8.00]
Total length of hospital stay [d], median [IQR]		3.00 [1.00, 15.00]	3.00 [1.00, 14.00]	3.00 [1.57, 16.10]	1.00 [1.00, 8.50]	–	11.00 [1.00, 16.00]
ISTH major bleeding, *n* (% [95% CI])		21 (30.4 [20.8, 42.1])	5 (17.2 [7.6, 34.5])	11 (40.7 [24.5, 59.3])	3 (37.5 [13.7, 69.4])	–	2 (40.0 [11.8, 76.9])
ISTH non-major bleeding, *n* (% [95% CI])		16 (23.2 [14.8, 34.4])	4 (13.8 [5.5, 30.6])	7 (25.9 [13.2, 44.7])	2 (25.0 [7.1, 59.1])	–	3 (60.0 [23.1, 88.2])
Stroke, *n* (% [95% CI])		7 (10.1 [5.0, 19.5])	2 (6.9 [1.9, 22.0])	4 (14.8 [5.9, 32.5])	1 (12.5 [2.2, 47.1])	–	0 (0.0 [0.0, 43.4])
Cerebral performance category (CPC) of survivors on hospital discharge	CPC 1, *n* (% [95% CI])	12 (66.7 [43.7, 83.7])	5 (71.4 [35.9, 91.8])	5 (83.3 [43.6, 97.0])	1 (50.0 [9.5, 90.5])	–	1 (33.3 [6.1, 79.2])
	CPC 2, *n* (% [95% CI])	4 (22.2 [9.0, 45.2])	0 (0.0 [0.0, 35.4])	1 (16.7 [3.0, 56.4])	1 (50.0 [9.5, 90.5])	–	2 (66.7 [20.8, 93.9])
	CPC 3, *n* (% [95% CI])	2 (11.1 [3.1, 32.8])	2 (28.6 [8.2, 64.1])	0 (0.0 [0.0, 39.0])	0 (0.0 [0.0, 65.8])	–	0 (0.0 [0.0, 56.1])
	CPC 4, *n* (% [95% CI])	0 (0.0 [0.0, 17.6])	0 (0.0 [0.0, 35.4])	0 (0.0 [0.0, 39.0])	0 (0.0 [0.0, 65.8])	–	0 (0.0 [0.0, 56.1])

CI, confidence interval; CPC, cerebral performance category; CPR, cardiopulmonary resuscitation; CT, computed tomography; ESC, European Society of Cardiology; ICU, intensive care unit; IQR, interquartile range; ISTH, International Society of Thombosis and Haemostasis; PaCO2, arterial partial pressure of carbon dioxide; PaO2, arterial partial pressure of oxygen; PaO2/FiO2, arterial partial pressure of oxygen to fractional inspired oxygen; PCDT, percutaneous catheter-directed treatment; PE, pulmonary embolism; RV, right ventricle; SAPS II score, simplified acute physiology II score; SOFA score, sequential organ failure assessment score; ST, surgical thrombectomy; SYS, systemic thrombolysis; VA-ECMO, veno-arterial extracorporeal membrane oxygenation. *First value measured at hospital admission

Main findings of this prespecified sub-analysis are as follows: (I) In-hospital mortality following prehospital SYS was high, with most suffering cardiac arrest, (II) in-hospital mortality was lower following PCDT compared to VA-ECMO or intrahospital SYS, though caution with interpretation must be taken due to lack of statistical comparison and risk of clinical selection bias, and (III) in-hospital mortality was similar for patients escalated to VA-ECMO and those not receiving further treatment, despite more major bleeding with VA-ECMO – though again caution with interpretation is warranted. The high proportion of patients receiving thrombolytic therapy based on clinical suspicion of PE highlights the difficulty of diagnosing PE in hemodynamically unstable patients or during cardiopulmonary resuscitation in the out-of-hospital setting and emphasizes the role of focused echocardiographic assessment by emergency medical personnel. Our study was not specifically designed to evaluate the initial diagnostic process (e.g. exclusion of patients in whom PE was refuted after initial suspicion). Further investigation of diagnostic and management algorithms that do not rely on computed tomography imaging are needed. Regarding outcomes, previous studies have described even higher mortality rates following prehospital SYS for acute PE [4, 5]. In a retrospective analysis from the French National Out-of-hospital Cardiac Arrest (RèAC) registry, Javaudin et al. reported outcomes of 246 patients suffering out-of-hospital cardiac arrest, in whom PE was confirmed after hospital admission [5]. The unadjusted 30-day survival rate was 16%, with just 10% of patients achieving a Cerebral Performance Category score of 1 or 2 among patients receiving thrombolysis during cardiopulmonary resuscitation. However, comparability is limited due to differences in baseline characteristics (e.g., median age 60.5 vs. 54.0 years) and management (e.g., VA-ECMO support in 9% vs. 47.8%) [5]. Interestingly, patients who received SYS during resuscitation had a lower 30-day mortality rate compared to controls without SYS in a weighted analysis [5]. Furthermore, risk of bleeding with SYS must also be weighed against potential benefit. Despite evidence of clinical benefit following SYS for high-risk PE in small studies, high bleeding rates are common [4]. We found more major bleeding with VA-ECMO without lower mortality in our cohort. Hence, benefit of mechanical circulatory support may be diminished by bleeding and its use discouraged following SYS. Encouragingly, major bleeding was similar despite large differences in duration of cardiopulmonary resuscitation (30 min) between VA-ECMO and intrahospital SYS patients, a result supported by similar findings in other studies [4]. Thus, as reflected by guidelines, current evidence can only support VA-ECMO as a rescue therapy and bridge to further reperfusion strategies for high-risk PE [2]. Randomized studies addressing mechanical circulatory support in the setting of high-risk PE and prehospital SYS are needed. Patients receiving PCDT, for which enthusiasm is currently mainly backed by evidence in intermediate-risk PE, had lower mortality than patients undergoing VA-ECMO or intrahospital SYS in our cohort. However, retrospective design, potential selection bias toward lower risk patients, and limited sample size are limitations. Consequently, these findings are only hypothesis-generating and must be interpreted with the utmost caution.

Overall, conducting large randomized controlled trials in this population poses a significant challenge and has yet to be done. Alternative data acquisition techniques that reduce bias will be required to circumvent difficulties with recruitment for randomized trials [3]. Lastly, there is a need to understand which patients benefit from prehospital SYS and which treatment (if any) should follow. Adequately powered randomized controlled trials are urgently required to improve dismal mortality rates for high-risk PE.

## Data Availability

The data are not publicly available due to ethical restrictions and legal constraints. Readers may contact the corresponding authors for reasonable requests for the data. De-identifed data may be provided after approval from the ethical review board.
